# Research on the spatiotemporal characteristics and influence mechanism of the integration of the health industry and tourism industry in the Chengdu-Chongqing economic circle of China

**DOI:** 10.3389/fpubh.2023.1234259

**Published:** 2023-09-13

**Authors:** Xuejun Chen, Ying Deng

**Affiliations:** ^1^Chong Qing Jiaotong University, Chongqing, China; ^2^People’s Government of Liangping Zhushan Township, Chongqing, China

**Keywords:** Chengdu-Chongqing economic circle, health tourism, spatiotemporal characteristics, influence mechanism, driving factor

## Abstract

**Introduction:**

Given the background of the “Healthy China” strategy and the aging population, the high-quality integration of the health industry and tourism industry is of great significance to meet the national health tourism needs and promote regional coordinated development.

**Methods:**

Taking the Chengdu-Chongqing economic circle as an example, this paper uses the coupling coordination degree method and geographic detectors to explore the spatiotemporal characteristics and influence mechanism of the integration of the health industry and tourism industry in the Chengdu-Chongqing region.

**Results:**

(1) The integration level of the Chengdu-Chongqing economic circle as a whole, four major regional sectors, and 36 cities is relatively low, and the regional differences in the degree of coupling coordination are relatively large, showing a three-level change. Overall, the spatial differentiation characteristics are high in the west and low in the east, radiating from the two nuclei to the periphery. (2) There are obvious regional differences in the spatial connection strength of the integration of the two major industries, which form a radial non-equilibrium structure with the Chengdu and Chongqing main cities as the connection centers. The overall spatial connection network structure is relatively loose, the point-degree centrality has the spatial distribution characteristics of dominated by two poles, led by multiple cores, and weak at the edges, and the division of cohesive subgroups is greatly affected by geographical factors and administrative divisions. (3) The temporal and spatial characteristics of the integration of the two industries are the result of the mutual coupling of market demand, industrial development, policy support, and economic development.

**Discussion:**

The high-quality integrated development of the health industry and the tourism industry is a complex systematic project, which requires the assistance of government departments at all levels and health tourism enterprises in the Chengdu-Chongqing economic circle to strengthen coordination and cooperation.

## 1. Introduction

At the sixth meeting of the Central Finance and Economics Commission on January 3, 2020, General Secretary Xi Jinping made a significant decision to advance the development of the Chengdu-Chongqing economic circle and establish an important growth pole for high-quality development. Since then, academic research has focused heavily on the region’s synergistic cooperation between Chengdu and Chongqing. The health industry and tourism industry are highly complementary and have natural coupling. In the process of integration, the industrial boundary is gradually blurred, the industrial chain is reconstructed and optimized, the original industrial value chain is expanded to another industry through crossover and penetration, the division of labor and collaboration between the health industry and tourism industry is transformed into the division of labor within the industry, the original elements, resources, products and services belonging to the health and tourism industries are continuously circulated, crossed, integrated and optimized, resulting in a superposition effect stronger than the original industry effect, and the integration of the two industries is a process of dynamic reorganization and dynamic optimization of resource elements. In the context of the “Health China” strategy, the aging population and COVID-19, the integration of the health industry with the tourism industry is of great practical significance. In this context, health tourism as an emerging form of tourism has received attention. Its role in health and wellness, medical care, and recreation is in line with the recreational needs of mass travelers at this stage and is an important breakthrough point to crack the dilemma of tourism development in the post-epidemic era, as well as an important way to promote the construction of the Chengdu-Chongqing economic circle and make up for the shortcomings of development.

By exploring the spatial and temporal characteristics and influence mechanisms of the integration of the health and tourism industries in the Chengdu-Chongqing economic circle, the reform performance of the Health and Tourism Commission and the Culture and Tourism Commission can be reflected a certain extent, which has important decision-making value for the creation of a demonstration zone for health and tourism and the high-quality integrated development of the industries and is of great significance for enhancing the comprehensive strength and competitiveness of the Chengdu-Chongqing economic circle, promoting coordinated regional development and forming a regional economic layout with complementary advantages and high-quality development. It is certain that, as the reform continues to advance in-depth, the boundary between the health and tourism industries will become increasingly blurred, the degree of integration between the two industries will deepen, and the integration environment, the supply of recreation tourism, the consumer market and the consumer concept will be significantly improved, which will have a positive effect on the development of health tourism in the Chengdu-Chongqing region, which also fully reflects the foresight and scientific nature of the Party Central Committee’s decision to make a major reform of the health and tourism system mechanism.

## 2. Review of the literature

Early research into the integration of the health and tourism industries began in the 1980s, but most of the existing studies focus on the coupled and coordinated development of the tourism industry with the cultural industry (1), ecological environment, urbanization (2), economic development (3), and rural revitalization (4). Only a few studies deal with the integration with the health industry (5). The connection between the health industry and the tourism industry has become a major topic for scholarly debate since the COVID-19 broke out in 2020 and mass travelers’ demand for health and wellness intensified. At present, the research results on the integration of the health industry and tourism industry are mainly focused on five aspects: integration mechanisms, integration products, integration evaluation, impact effects, and driving factors.

### 2.1. Integration mechanisms

The integration mechanism of the health industry and the tourism industry mainly includes the integration dynamics, the integration mode and the integration path. In terms of integration dynamics, both the health and tourism industries involve multiple industries and sectors and are influenced by multiple drivers. Therefore, the integration of the health industry and tourism industry can be summarized as internal and external dynamics, with internal dynamics mainly being the upgrading of consumer demand, the pursuit of corporate interests, market demand, industrial development needs and industrial competition (6), and external dynamics mainly being the top-level design of the government, the laws of market development and the level of economic development (7). In terms of integration mode, the two industries have basically formed “health + tourism” nesting and “tourism + health” nesting mode (8), “tourism resources for the older adult” and “older adult institutions interaction” mode in the process of mutual integration (9). In terms of integration paths, the cluster development path (10), the “four paths,” namely resource integration, technology integration, market integration and functional integration (11), and the “two paths,” namely extensional integration and penetration integration (8), are the common paths for the integration of the health and tourism industries.

### 2.2. Integration products

The integration of the health industry with the tourism industry eventually resulted in the formation of new tourism products such as health tourism (12), sports and wellness tourism (13), medical and wellness tourism (14), rural ecological and wellness tourism (15), hot springs and wellness tourism (16), forestry and wellness tourism (17). In particular, Chinese medicine played an important role in the fight against COVID-19, and China attaches great importance to the development of Chinese medicine heritage, so Chinese medicine and health tourism may become a crucial topic, topic of high importance, or a common point of discussion for future research on the integration of the two industries. Foreign scholars mainly focus on specific forms of medical tourism, spa tourism, forest tourism, yoga tourism and religious tourism, with a predominance of studies devoted to medical tourism. In the case of medical tourism, it has both positive and negative effects on the physical and psychological well-being of travelers (18). In the case of spa tourism, forest tourism and yoga tourism, their positive physiological, psychological and economic and social effects on tourists are the focus of research by foreign scholars (19).

### 2.3. Integration evaluation

There are two main approaches to the evaluation of the integration of the health industry and the tourism industry: one is to qualitatively analyze the problems of industrial integration by describing the current situation of industrial integration (20); the other is to quantitatively study the dynamics of industrial integration using mathematical and theoretical methods, etc. The former is the common evaluation method used by academia, and only a few scholars have used the Moran index, grey correlation method and topological analysis to quantitatively evaluate the integration status of the two industries. Although most of the qualitative studies on the evaluation of the integration of the health industry and the tourism industry have been conducted, quantitative empirical research is the future trend for the evaluation of the integration of the two industries. Academics can use coupling and coordination models, input–output models, optimal entropy method models, data envelopment analysis and other research methods to quantitatively measure the degree of integration, spatial characteristics, industrial viscosity, industrial efficiency and influence mechanisms of the health industry and the tourism industry.

### 2.4. Impact effects

The impact of the integration of the health industry and the tourism industry is reflected at the level of both the individual tourist and the tourist destination, specifically in terms of individual welfare effects, economic effects and social effects. The welfare effects of the integration of the two include positive effects on physical, mental and spiritual health, improved quality of life and a greater sense of well-being (21). The economic effect is reflected in promoting local economic development, increasing foreign exchange earnings and maintaining the national trade balance, driving market consumption, raising wage levels and increasing employment. The social effect is reflected in the enhancement of world culture and knowledge exchange, and the promotion of social prosperity (22). However, the integration and development of the health and tourism industries can also bring about some negative effects. For example, it increases the burden on the local environment, living and medical public facilities, raises the price of housing and consumption levels, fierce competition in the industry, damages the ecological environment and makes it difficult to protect the rights and interests of tourists.

### 2.5. Driving factors

With the changes in society, economic, social and technological factors have driven the emergence and rapid development of a new industry that integrates the health and tourism industries, namely the health tourism industry. In terms of economic factors, the integrated development of the world economy has greatly facilitated economic and social exchanges between peoples. International pensioners and medical tourists generate huge foreign exchange earnings from tourism for destination countries, directly driving governments to introduce facilitative visa policies (23). In terms of social factors, as income levels rise and consumer attitudes upgrade, people are spending more on maintaining health and preventing disease. In particular, consumers’ healthy lifestyles and consumption concepts have been reinforced by the impact of the COVID-19, and health tourism has become a market consumption trend (24). Digital Free Tourism is a growing economic trend and that the phenomenon of digital disconnection is beginning to be a peremptory need for tourists, health tourism is gradually becoming a temporary escape and alternative to technological devices (25). In terms of technological factors, transformative innovations in science and technology have greatly enhanced the convenience and speed of wellness tourism consumption. Faster and more economical modes of transport such as motorways, high-speed railways and aeroplanes have reduced travel times and costs for travelers (26). Modern information technology revolutions such as 5G, big data, cloud computing and blockchain lead to consumer upgrades, greatly improving the convenience and comfort of health tourism consumption (27). The innovation and development of mobile health applications in the health industry not only provide convenience for health tourists, but also focus on protecting user privacy and personal data (28).

Overall, domestic and foreign scholars mainly study the integration of tourism industry with cultural industry, sports industry, and agriculture, but there are few research results on the integration of tourism industry with health industry. Academic research on the integration of the health and tourism industries has focused on five areas: integration mechanisms, integration products, integration evaluation, impact effects and driving factors, with increasingly in-depth research into such sub-sectors as older adult tourism, medical tourism, health tourism, and related research gradually moving towards maturity. The above research results mostly qualitatively discuss the basic theories of the integration of the health industry and tourism industry, but overlook the quantitative research related to the evaluation of the integration of the health industry and tourism industry. In particular, there is a lack of practical research that explores the coupling between the health and tourism industries, their spatial effects and impact mechanisms from a geographical perspective and at a regional level. In short, the lag in theoretical and practical research has seriously constrained the healthy development of the health tourism industry, and it is urgent to strengthen relevant research.

In view of this, the Chengdu-Chongqing economic circle is chosen as the study area from a geographic perspective. The coupling coordination degree method, modified gravity model, social network analysis method, and geographic probe are used to analyze the spatial and temporal characteristics of the integration of the health industry and tourism industry as well as their influence mechanisms. This is crucial for broadening the geographic study of the two industries’ integration in the Chinese context, understanding the regional trend of their integration, and improving the industrial layout and regional synergistic development.

## 3. Study design

### 3.1. Study area overview

**Figure 1:**
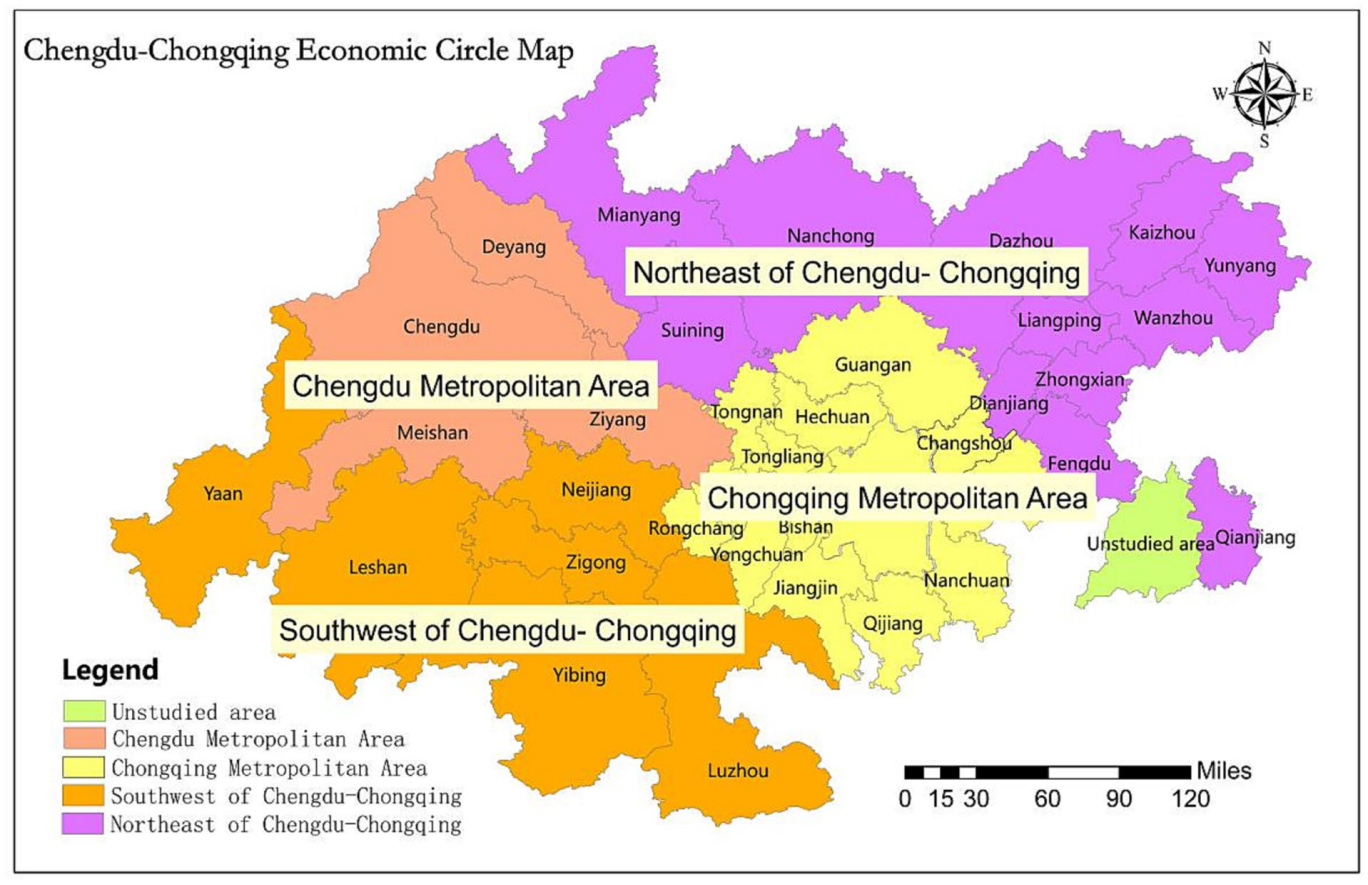
The Chengdu-Chongqing economic circle map.

With a total area of 185,000 square kilometers and a gross regional product of almost 6.6 trillion yuan in 2020, the Chengdu-Chongqing economic circle includes 15 cities in Sichuan Province and 29 districts in Chongqing. However, because it is challenging to remove the data from Kaizhou, Yunyang, Mianyang, Dazhou, and Ya’an, which are not part of the planning area but have a little impact on the overall results, they are included in the study together with the cities where they are located. Furthermore, the Chongqing Yuzhong District, Yubei District, Nanan District, Banan District, Beibei District, Jiangbei District, Jiulongpo District, Shapingba District, and Dadukou District are integrated into the main urban area of Chongqing. Therefore, the study area of the Chengdu-Chongqing economic circle includes 15 cities in Sichuan province and 21 districts in Chongqing, totaling 36 study cities (29). It is divided into four geographical segments: Chongqing metropolitan area, Chengdu metropolitan area, northeastern Chengdu-Chongqing region, and southwestern Chengdu-Chongqing region. The planning scope is shown in Figure 1.

### 3.2. Indicator selection and data sources

#### 3.2.1. Indicator selection

The indicators for gauging the level of development in the health and tourism industries are intricate and complex. They span a wide range, but in general, they can be distilled into three categories—people, money, and materials—which are specifically expressed as market demand, industry scale, and reception capacity (30). The evaluation index system of the integration degree of the health industry and tourism industry is constructed from the three aspects of market demand, industry scale, and reception capacity based on the findings of the existing research (see Table 1).

**Table 1:** Evaluation index system of the integration degree of health industry and tourism industry.

Industry Category	Primary indicators	Secondary indicators	Indicator Type
Tourism Industry	Industry Scale	Total Tourism Revenue	Positive
		Total tourist arrivals	Positive
	Market Demand	*Per capita* tourism spending	Positive
		*Per capita* disposable income of urban residents	Positive
		*Per capita* disposable income of rural residents	Positive
		Gross regional product *per capita*	Positive
	Reception Capacity	Travel Agency	Positive
		A-class scenic spot	Positive
		Star Hotels	Positive
Health Industry	Industry Scale	Total revenue of health care institutions	Positive
		Total number of consultations	Positive
	Market Demand	Number of health checkups	Positive
		*Per capita* health care expenditure of urban residents	Positive
		*Per capita* health care expenditure of rural residents	Positive
	Reception Cpacity	Number of medical and health institutions	Positive
		Number of beds in health institutions	Positive
		Number of health technicians	Positive

#### 3.2.2. Data source

As the study area has gone through the development process from “Chengdu-Chongqing City Cluster” to “the Chengdu-Chongqing economic circle,” and the regional scope has not changed, the period of 2016–2020 is chosen as the study period. The research data were primarily gathered from the 2016–2020 Sichuan Provincial Statistical Yearbook, Sichuan Provincial Health Statistical Yearbook, Chongqing Municipal Statistical Yearbook, and Chongqing Municipal Health Statistical Yearbook. Some of the missing data were supplemented by linear interpolation or the mean value method. In addition, the urban prime distances were calculated by the ArcGIS software, and the data on the number of health tourists, health tourism income, and tourism enterprises were obtained by conversion (31), since there are no direct statistics on the number of health tourism and tourism enterprises. Among them, the number of health tourists = the number of national health tourists × the proportion of tourists from various regions to the total number of tourists in the country, the number of national health tourists = proportion of urban residents’ health tourists × number of domestic tourists from urban residents + proportion of rural residents’ health tourists × number of domestic tourists from rural residents; health tourism income = total national health tourism income × the ratio of the total tourism income of each region to the total tourism income of the country, total national health tourism income = number of urban residents who travel for health care × *per capita* expenditure on urban residents’ health and recuperation tourism + number of rural residents’ health and recuperation tourism × *per capita* expenditure on health and recuperation tourism for rural residents; number of tourism enterprises = number of travel agencies + number of A-level scenic spots + number of star rated hotels.

### 3.3. Research methodology

#### 3.3.1. Coupling coordination degree method

The tourism and health industries are logically related, and the coupling coordination degree model can more effectively depict the degree of coupling between the two industries (32). The health and tourism industries were viewed in this paper as two mutually coupled subsystems, and by using the entropy value method to determine the weight of indicators, a coupling coordination degree model was built that could reflect the degree of coupling and coordination between the two industries in the Chengdu-Chongqing economic circle. The coupling coordination degree approach had the following steps (33):

1. Calculation of coupling

(1) $$C=2\times \sqrt{\frac{u_{1}\times u_{2}}{{\left(u_{1}+u_{2}\right)}^{2}}}$$

1. Calculating the composite coordination index

(2) $$T=\alpha u_{1+}\beta u_{2}$$

1. Calculation of coupling coordination

(3) $$D=\sqrt{C\times T}$$

where C is the coupling degree of the two subsystems, T is the comprehensive coordination index of the two subsystems, and D is the coupling coordination degree. u_1_, u_2_ are the comprehensive development indices of the health industry subsystem and the tourism industry subsystem. α, β are undetermined coefficients, considering that the health industry and tourism industry are equally important, so α = β = 0.5.

In addition, in order to more clearly reflect the coupling and coordination of the health industry and tourism industry in the Chengdu-Chongqing economic circle, we referred to Liao Chongbin’s (34) classification criteria for the degree of coupling and coordination.

#### 3.3.2. Modified gravitational model

The spatial connection value can reflect the radiation effect of a core city on adjacent cities. The gravitational model is a useful tool for quantifying the spatial interaction of an urban system. However, the original gravitational model had to be improved by including the coupling coordination degree in order to appropriately reflect the geographical linkage strength of the health industry and tourism industry integration in the Chengdu-Chongqing economic circle (35).


(4) $$R_{ij}=k\cdot \frac{\sqrt[{3}]{P_{i}G_{i}D_{i}}\sqrt[{3}]{P_{j}G_{j}D_{j}}}{{\left(d_{ij}\right)}^{2}},\;k=\frac{D_{i}}{D_{i}+D_{j}}$$

(5) $$R_{i}=\overset{n}{\underset{j=1}{\sum }}R_{ij}$$

$R_{ij}$ denotes the spatial linkage strength of the coupling coordination between cities i and j. $R_{i}$ denotes the spatial linkage potential value of city i, which is the sum of the amount of spatial linkage between city i and other cities. The larger its value, the stronger the coupled and coordinated spatial linkage between city i and other cities. $P_{j}$ denotes the number of the urban population in cities i and j; $G_{i},G_{j}$ denotes the gross regional product of cities i and j; $D_{i}$ , $D_{j}$ denotes the coupling coordination of cities i and j; and $d_{ij}$ denotes the prime distance between cities i and j. k is the modified empirical constant, which is expressed as the proportion of the coupling coordination of a city to the sum of the coupling coordination of two cities.

#### 3.3.3. Social network analysis method

The social network analysis approach, which has two main research perspectives—the overall network and the individual network—is a key method for analyzing the nodes and structural aspects of spatial networks based on the relationship data between nodes (36). In this study, the Chengdu-Chongqing economic circle was viewed as a sizable network, with the cities and the cities’ spatial connections being viewed as network nodes and inter-node links, respectively. The spatial linkage association matrix of the 36 cities calculated by the above gravity model was binarized using the mean-average method. Meanwhile, with the help of the UCINET software, the indicators of network density, centrality, and cohesive subgroups in the social network analysis method were used to explore the spatial connection network characteristics of the Chengdu-Chongqing economic circle.

#### 3.3.4. Geodetector

Geodetector is a statistical method that detects spatial differentiation and reveals the driving factors behind it, including four types of detectors—factor detection, interaction detection, ecological detection, and risk detection—and has the advantage of immunity to the covariance of independent variables and is more reliable than classical regression (37). With the measured integration degree as the dependent variable and 10 detection factors as the independent variables, factor detection and interaction detection in the geographic detectors were used to analyze the factors influencing the level of integration development of the health industry and tourism industry in the Chengdu-Chongqing economic circle.

## 4. Spatial and temporal characteristics of the integration of the health industry and tourism industry

### 4.1. Chronological evolution characteristics

After standardizing the raw data, Eq. (1) to (3) were applied to determine the degree of cooperation between the health and tourism industries in the Chengdu-Chongqing economic circle from 2016 to 2020 (see Table 2).

**Table 2:** Coupling and coordination of health industry and tourism industry.

City	2016	2017	2018	2019	2020	Average value	City	2016	2017	2018	2019	2020	Average value
Chengdu	0.599	0.621	0.649	0.682	0.663	0.643	Qijiang	0.146	0.159	0.167	0.177	0.178	0.165
Zigong	0.229	0.243	0.256	0.271	0.267	0.253	Dazu	0.143	0.155	0.168	0.187	0.197	0.170
Luzhou	0.275	0.290	0.302	0.314	0.308	0.298	Qianjiang	0.121	0.133	0.147	0.156	0.162	0.144
Deyang	0.249	0.261	0.279	0.293	0.281	0.273	Longevity	0.151	0.161	0.171	0.185	0.197	0.173
Mianyang	0.303	0.316	0.329	0.342	0.339	0.326	Jiangjin	0.178	0.190	0.199	0.209	0.210	0.197
Suining	0.243	0.253	0.263	0.277	0.277	0.263	Hechuan	0.156	0.170	0.183	0.200	0.206	0.183
Neijiang	0.222	0.236	0.249	0.262	0.264	0.247	Yongchuan	0.178	0.189	0.200	0.219	0.225	0.202
Leshan	0.278	0.291	0.303	0.316	0.315	0.301	Nanchuan	0.151	0.144	0.163	0.169	0.175	0.160
Nanchong	0.304	0.318	0.332	0.344	0.349	0.329	Bishan	0.151	0.162	0.174	0.197	0.200	0.177
Meishan	0.243	0.241	0.257	0.267	0.264	0.255	Tongliang	0.141	0.154	0.163	0.182	0.196	0.167
Yibin	0.274	0.299	0.314	0.328	0.331	0.309	Tongnan	0.128	0.141	0.148	0.158	0.175	0.150
Guang’an	0.242	0.254	0.261	0.270	0.261	0.257	Rongchang	0.135	0.149	0.160	0.175	0.189	0.162
Dazhou	0.270	0.265	0.271	0.288	0.291	0.277	Liangping	0.138	0.150	0.167	0.192	0.201	0.169
Ya’an	0.210	0.221	0.233	0.244	0.243	0.230	Fengdu	0.110	0.120	0.134	0.147	0.155	0.133
Ziyang	0.219	0.226	0.236	0.238	0.239	0.232	Dianjiang	0.146	0.159	0.171	0.187	0.199	0.173
Chongqing Main City	0.445	0.478	0.495	0.511	0.510	0.488	Zhongxian	0.128	0.141	0.160	0.174	0.182	0.157
Wanzhou	0.196	0.207	0.214	0.224	0.229	0.214	Kaizhou	0.142	0.154	0.162	0.173	0.177	0.161
Fuling	0.175	0.191	0.201	0.212	0.224	0.201	Yunyang	0.116	0.131	0.147	0.167	0.175	0.147
Chengdu Metropolitan Area	0.374	0.390	0.419	0.449	0.441	0.415	Chongqing Metropolitan Area	0.285	0.316	0.342	0.372	0.385	0.340
Southwest Chengdu -Chongqing	0.279	0.310	0.339	0.369	0.370	0.333	Northeast Chengdu-Chongqing	0.284	0.307	0.336	0.367	0.377	0.334
Chengdu-Chongqing Economic Circle	0.611	0.640	0.671	0.703	0.694	0.664							

Table 2 shows that the degree of coupling and coordination for the entire Chengdu-Chongqing economic circle was fluctuating and growing at a rate of 13.7% over the course of 5 years, peaking at 0.703 in 2019. The degree of coupling and coordination across the four main regional sectors was also varying and growing, with the Chengdu metropolitan area’s coupling degree being significantly better than that of the other three sectors at a growth rate of 18% over 5 years. With growth rates of 35.3, 32.6, and 32.5%, respectively, during the past 5 years, the coupling coordination of the Chongqing metropolitan area, northeastern Chengdu-Chongqing region, and southwest Chengdu-Chongqing region was essentially flush. Although there was a fluctuating upward trend in the degree of coupling and coordination between the health and tourism industries in the Chengdu-Chongqing economic circle, the impact of the epidemic caused a decline in the level of coupling and coordination in some of these hubs in 2020. Except for the Chengdu and Chongqing main city, the integration development levels of other cities in the economic circle were low. Most of them were less than 0.3. The integration of the health industry and tourism industry in Chengdu was the best. Significantly better than other regions, the coupling coordination degree grew from 0.599 in 2016 to 0.663 in 2020 with a growth rate of 10.7%. It ranked first overall, which was related to the health tourism resource endowment of Chengdu. With a coupling coordination degree rising from 0.445 to 0.510 and a development rate of 14.6%, Chongqing main city was second only to Chengdu in terms of the integration effect of the health and tourism industries. With a coupling coordination degree of 0.155 in 2020, less than one-fifth of Chengdu, the integration effect of the health industry and tourism industry in Fengdu was the worst, and the backward development level of the two industries prevented the high-quality integration development of the two sectors. Overall, the degree of coupling coordination in the Chengdu-Chongqing economic circle, the four geographical segments, and the 36 cities in 2020 increased compared with that of 2016, but the level of coupling coordination was still low, and there is still potential for improvement.

### 4.2. Spatial divergence characteristics

**Figure 2:**
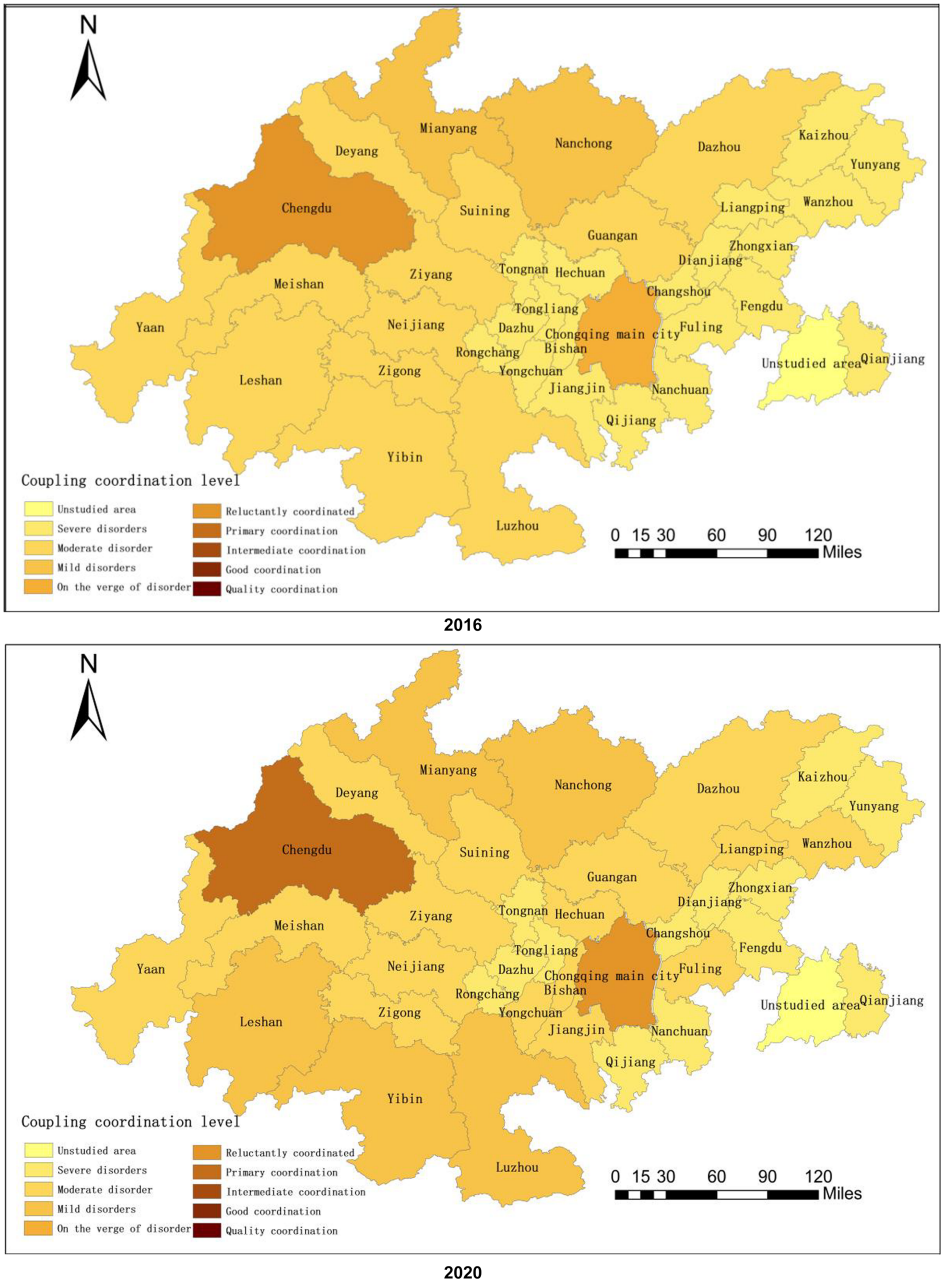
Spatial distribution of health industry and tourism industry coupling coordination.

The two industries exhibited significant differences in their levels of coupling and coordination as well as the general spatial divergence features of high in the west and low in the east, radiating from the two cores to the periphery, as shown by the spatial divergence map (see Figure 2).

Overall, the Chengdu-Chongqing economic circle’s coupling coordination was primarily distributed within the range of (0.6, 0.8), which showed primary coordination from 2016 to 2018, intermediate coordination in 2019, and a return to primary coordination in 2020 as a result of the epidemic’s effects. It is clear that even though the Chengdu-Chongqing economic circle as a whole has reached a state of coordination, effective action is still required to address the adverse effects of environmental changes on the integration of the two industries. From the perspective of each geographical segment, the coupling coordination of the four major segments was concentrated within (0.2, 0.5), and the coordination status of the two major industries gradually improved, with the southwestern Chengdu-Chongqing region, the northeastern Chengdu-Chongqing region, and the Chongqing metropolitan area developing from moderate disorder to mild disorder and the Chengdu metropolitan area developing from mild disorder to near disarray. From each city’s perspective, the coupling coordination degree of the 36 cities in 2016 was primarily distributed within the range of (0.1, 0.6). The severe disorder level included 20 cities in the Chongqing area, and the moderate disorder level included 12 cities in the Sichuan area. This indicated that more than half of the cities were in these stages, with a low level of integration between the two regions. The mild disorder level comprised two cities, Mianyang and Nanchong, while Chongqing main city was on the verge of the disorder level, and only Chengdu reached barely coordinated. The coupling coordination degree of the 36 cities in 2020 was primarily distributed within (0.1, 0.7). The severe dissonance level included 13 cities in the Chongqing area, the moderate dissonance level included 16 cities, and the mild dissonance level included Luzhou, Mianyang, Leshan, Nanchong, and Yibin. It can be seen that more than half of the cities were still in the severe and moderate dissonance stages, and the integration degree of the health industry and tourism industry was still low. In short, only the Chengdu-Chongqing economic circle as a whole, Chengdu and Chongqing main city reached a state of coordination. The four major geographical segments and the remaining 34 cities were all in a state of dissonance with low levels of coordination between the two major industrial couplings and very obvious intra-regional differences.

Additionally, the Chengdu-Chongqing economic circle’s level of coupling coordination could be roughly divided into three tiers. The regions and cities in intermediate coordination and primary coordination and those that were barely coordinated were classified as the first tier, the regions and cities on the verge of dissonance and were mildly disordered were classified as the second tier, and the regions and cities in moderate dissonance and severe dissonance were classified as the third tier. According to the ranking of the twin-city economic circle’s coupling coordination in the Chengdu-Chongqing region in 2020, the first echelon was made up of the Chengdu-Chongqing economic circle as a whole, Chengdu and Chongqing main city. The second echelon was made up of the four major geographical segments and five cities. The third echelon contained nine cities in the Sichuan region, such as Zigong and Deyang, and 20 cities in the Chongqing region, such as Qijiang and Dazu. In general, the gap between the three echelons is fairly obvious. The first echelon should act as a source of radiation in the health and tourism industries and strengthen resource sharing with the dysfunctional areas. The second echelon should strengthen regional synergy to improve the overall coupling and coordination of the Chengdu-Chongqing economic circle, and simultaneously, capital investment in the third echelon should be increased.

### 4.3. Spatial linkage network characteristics

In order to clarify each city’s place and function in the coupling and coordination of the spatial connection network as well as to further examine the spatial relationship between the integration of the health industry and tourism industry in the Chengdu-Chongqing economic circle, the modified gravity model and social network analysis method were used to explore the spatial connection strength characteristics and spatial connection network characteristics of the integration of the health industry and tourism industry.

#### 4.3.1. Spatial connection strength characteristics

**Figure 3:**
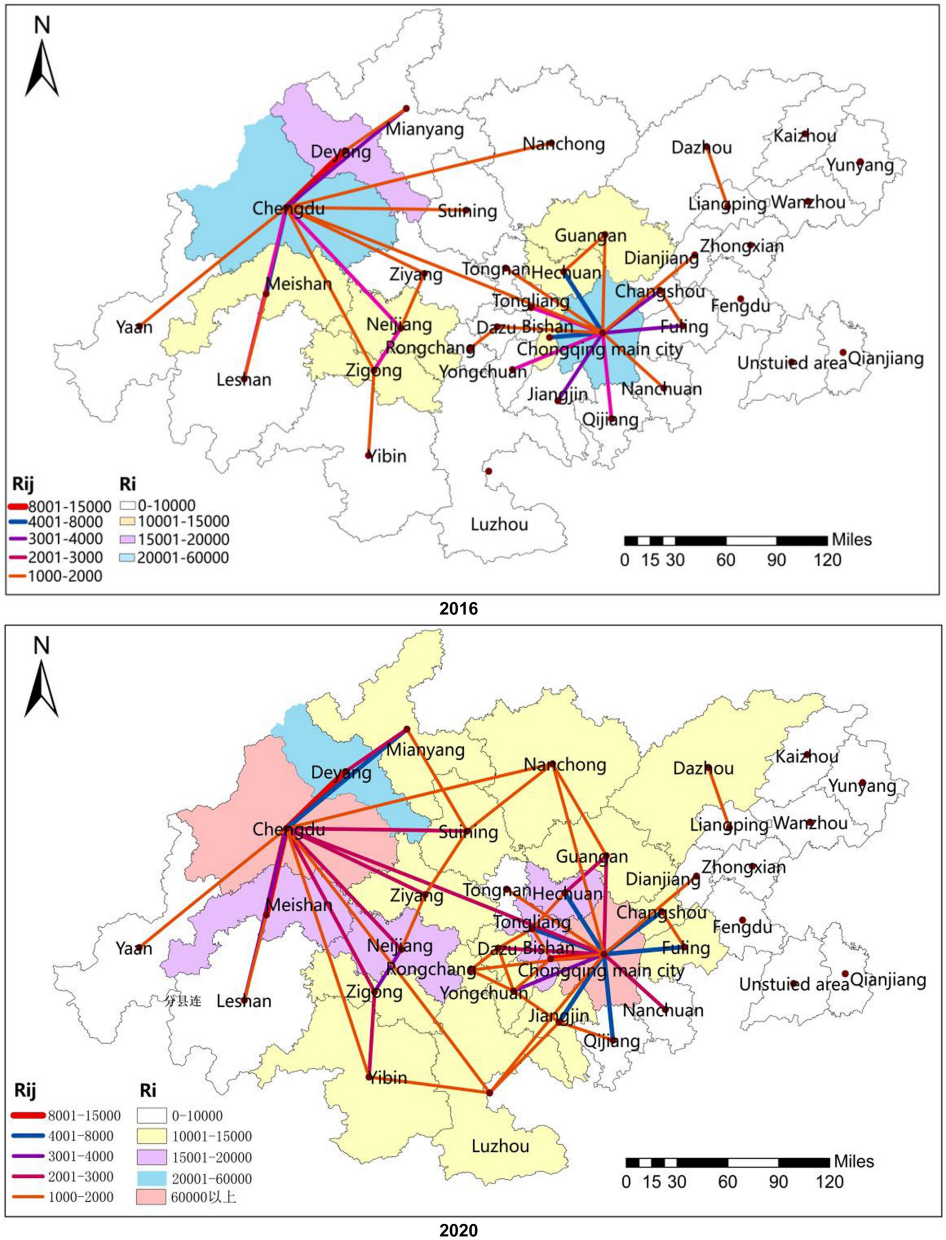
Spatial linkage intensity of health industry and tourism industry integration.

Equations (4) and (5) were used to compute the spatial connection strength of the integration of the two major industries in 2016 and 2020. The ArcGIS software was then used to depict the spatial connection strength of the cities with results larger than 1,000 (see Figure 3). In general, the regional differences in the spatial connection strength of the integration of the two major industries are obvious. The Chengdu and Chongqing main city established a radial non-equilibrium structure broadly described by the geographical distribution of two cores high, central weak, and northeast low. Ya’an, which is situated in the southwest of Chengdu-Chongqing, had very poor spatial connections with the majority of the cities, such as Qianjiang, Yunyang, and Kaizhou. These cities serve Chengdu and Chongqing main city are situated on the periphery of the Chengdu-Chongqing economic circle. It is quite simple to fall into the marginal area trap here, since other key cities had less radiation and were unable to integrate into the Chengdu-Chongqing economic circle’s spatial coupling and coordination network. On the border of the two main administrative regions are Tongnan and Rongchang. Despite being near Sichuan, they had nothing in common with the coupling and coordination spaces of the Sichuan cities. During construction, a depression in the bordering area formed, showing that the administrative division boundaries of the bordering area still exist, and Sichuan and Chongqing have not yet established a coupling, coordination, and co-governance mechanism for the cities in the bordering area. Therefore, these two types of cities should actively seek cross-regional cooperation in follow-up development and strive to get out of the marginal area trap and depression in the bordering area (38).

#### 4.3.2. Spatial linkage network characteristics

**Figure 4:**
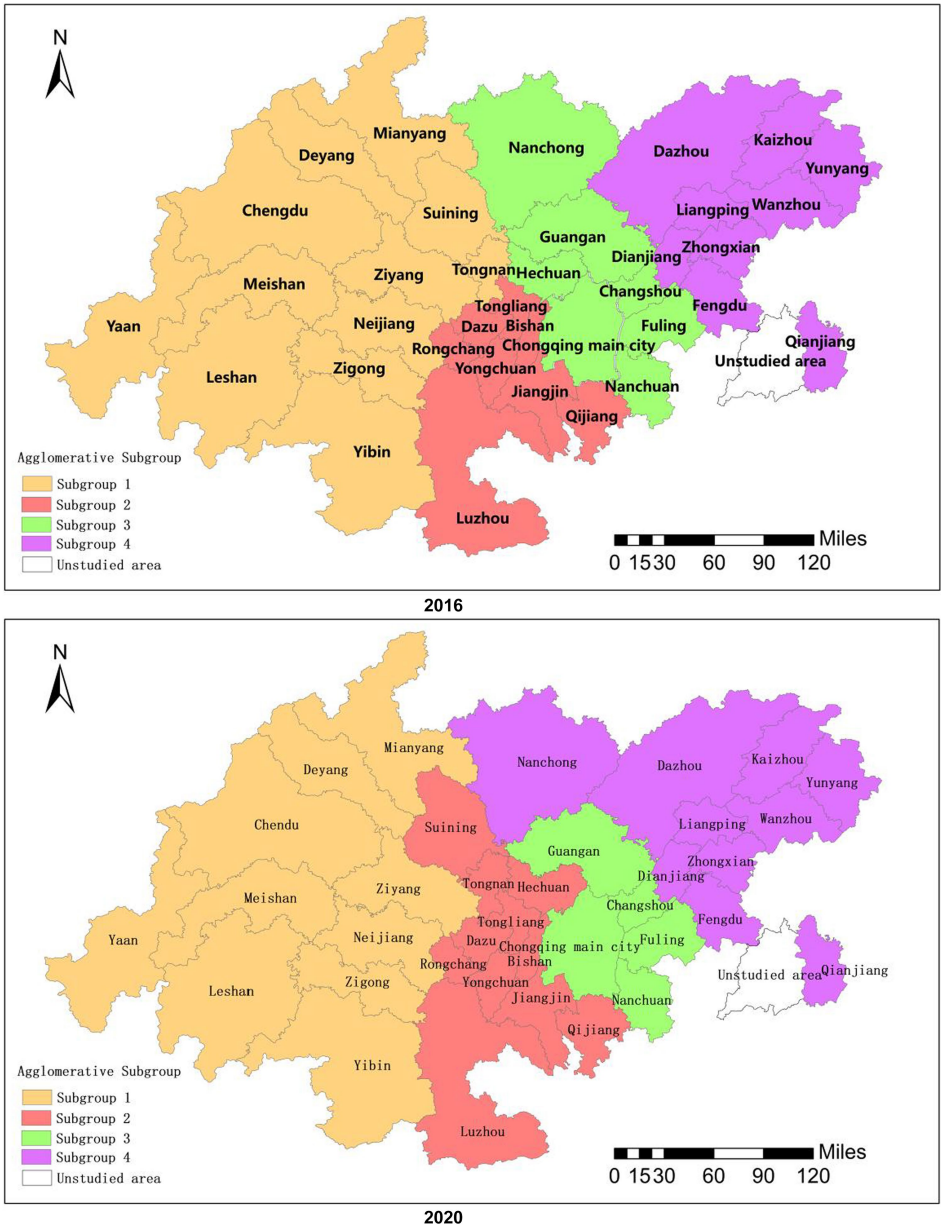
Spatial distribution of cohesive subgroups.

From the perspective of network density, the overall network densities of the two major industries in 2016 and 2020 were 0.238 and 0.240, respectively, indicating that the overall spatial connection network was relatively loose. In this network, the Chengdu and Chongqing main city have always been at the core and have the most complicated connections with other cities. Most cities, such as Ya’an, Yunyang, and Qianjiang, are at the edge of the network and have weaker connections with other cities. It can be seen that there was significant heterogeneity in the spatial connection network of the integration of the two industries, which showed typical scale-free characteristics (39). From the perspective of network centrality, the point-degree centrality of the Chengdu and Chongqing main city was significantly higher than that of other regions. The two occupied an absolute core position in the coupling and coordination spatial connection network and had the greatest degree of agglomeration and regional influence. There were significant regional differences and coupling coordination effects with the other 34 cities. In terms of points, Ziyang, Suining, Rongchang, Dazu, Dianjiang, Liangping and other cities improved their centrality. In 2020, they joined Neijiang, Guang’an, Nanchong, and Dazhou to form a secondary linkage center and a multi-level “small twin core” to guide the integration and growth of the cities in the spatial linkage network. Because they are less centralized and situated outside the research region, the cities of Ya’an, Qijiang, and Nanchuan were only marginally impacted by the spatial linking effect of the core cities. In general, the spatial distribution characteristics of the cities in the twin-city economic circle of the Chengdu-Chongqing region were bipolar dominance, multi-core leading, and weak at the edge. From the perspective of cohesive subgroups, the spatial linkage network of the health industry and tourism industry integration formed four cohesive subgroups at both points in time (see Figure 4), and the division was influenced by geographical factors and administrative divisions. The majority of the cities belonged to the same cohesive grouping, such as Chengdu, Deyang, Meishan, and Ziyang in subgroup 1, even though certain cities had undergone changes in the course of development. These cities have a more regular circulation of resources, information, and other components, and they have the building blocks to evolve into a community of coordinated regional development. In order to promote the high-quality integrated development of the health industry and tourism industry, we should actively push the boundaries of administrative divisions, strengthen cross-regional cooperation, promote the construction of regional coordinated development communities, and encourage regional linkage development and urban interconnection.

## 5. Influence mechanism of the integration of the health industry and tourism industry

### 5.1. Influencing factor index selection

The combination of regulatory support, economic development, market demand, and industrial development has led to the temporal and spatial evolution features of the integration of the health industry and tourism industry in the Chengdu-Chongqing economic circle (40). An index system of factors influencing the integration of the health industry and tourism industry was built using existing research findings as well as the traits of the health industry and the tourism industry and adhering to the principles of reliability, availability, and subjectivity of the index data (see Table 3).

**Table 3:** Health industry and tourism industry integration influence factor index system.

Influencing Factors	Measurement indicators	Detection factor
Policy Support	Total social fixed asset investment	$X_{1}$
	Local general public budget expenditure	$X_{2}$
Economic Development	Share of tertiary sector in GDP	$X_{3}$
	GDP *per capita*	$X_{4}$
Market Demand	Number of health tourists	$X_{5}$
	Total retail sales of social consumer goods	$X_{6}$
	*Per capita* health tourism consumption expenditure	$X_{7}$
Industry Development	Health tourism revenue	$X_{8}$
	Total revenue of health care institutions	$X_{9}$
	Number of tourism enterprises	$X_{10}$

### 5.2. Analysis of influencing factors

The results from factor detection indicated (see Table 4) that the four dimensions of policy support, economic development, market demand, and industrial development all had statistically significant effects on the spatial differentiation of the degree of integration between the health and tourism industries. The average influences of the influencing factors on the spatial differentiation of the degree of integration of the two industries in descending order were 0.980 for the total income of medical and health institutions, 0.961 for the investment in fixed assets of the whole society, 0.955 for the total retail sales of consumer goods, 0.941 for the general public budget expenditure, 0.936 for health tourism revenue, 0.935 for the number of health tourists, 0.820 for the number of tourism enterprises, 0.815 for the proportion of the tertiary industry in the GDP, 0.680 for *per capita* GDP, and 0.597 for *per capita* health tourism consumption expenditure. The factor detection means of the four major influencing factors from large to small were policy support (0.951), industrial development (0.912), market demand (0.829), and economic development (0.748). In general, each factor had an impact on the spatial differentiation of the degree of integration between the health industry and the tourism industry, but the degree of impact varied. Policy support was the dominant factor, industrial development was an important factor, and market demand and economic development were general factors.

**Table 4:** Factor detection results.

Detection factor	2016		2017		2018		2019		2020		Average value	
	q	Sort	q	Sort	q	Sort	q	Sort	q	Sort	q	Sort
$X_{1}$	0.944	4	0.966	4	0.966	3	0.970	2	0.957	2	0.961	2
$X_{2}$	0.941	5	0.983	1	0.929	6	0.930	6	0.924	5	0.941	4
$X_{3}$	0.941	5	0.878	7	0.868	7	0.693	9	0.694	8	0.815	8
$X_{4}$	0.671	9	0.681	9	0.699	9	0.708	8	0.639	9	0.680	9
$X_{5}$	0.922	7	0.927	6	0.944	5	0.939	5	0.942	4	0.935	6
$X_{6}$	0.950	3	0.950	5	0.979	1	0.945	3	0.950	3	0.955	3
$X_{7}$	0.608	10	0.613	10	0.625	10	0.578	10	0.560	10	0.597	10
$X_{8}$	0.970	2	0.975	3	0.966	4	0.945	4	0.826	6	0.936	5
$X_{9}$	0.979	1	0.980	2	0.978	2	0.986	1	0.976	1	0.980	1
$X_{10}$	0.844	8	0.839	8	0.814	8	0.821	7	0.781	7	0.820	7

To study the interaction mechanism of the spatial differential of the two major sectors, the top five elements with average influence were chosen (see Table 5). The detection outcomes demonstrated that the interaction of the two variables could enhance the interpretation of the spatial variations in the degree of integration between the two industries, producing a complementary enhancement impact of 1 + 1 > 2. The biggest explanatory power and greatest impact on the spatial difference in the degree of integration between the health industry and the tourism industry were provided by the interaction between the total income of medical and health institutions ( $X_{9}$ ) and health tourism revenue ( $X_{8}$ ). The influence had a much greater impact than how the other components interacted. Despite the relatively small impact of health tourism revenue in single-factor detection, the interaction with the other four factors had an explanatory power of 99%, demonstrating that the regional distribution pattern of integration degree was primarily created by the interaction between health tourism revenue and other factors. It is clear that the result of the interaction between each factor in the Chengdu-Chongqing economic circle was the spatial difference in the level of integration between the health industry and the tourism industry. The influence of many elements will need to be taken into account in the future when maximizing the geographical distribution pattern of the degree of integration of the two major industries in the Chengdu-Chongqing region.

**Table 5:** Interaction detection results.

Interaction Factor	2016		2017		2018		2019		2020		Average value	Sort
	q	Type	q	Type	q	Type	q	Type	q	Type		
$X_{1}\cap X_{2}$	0.993	DE	0.988	DE	0.989	DE	0.988	DE	0.985	DE	0.9886	8
$X_{1}\cap X_{6}$	0.958	DE	0.986	DE	0.984	DE	0.987	DE	0.984	DE	0.9798	10
$X_{1}\cap X_{8}$	0.996	DE	0.995	DE	0.996	DE	0.996	DE	0.976	DE	0.9918	4
$X_{1}\cap X_{9}$	0.993	DE	0.988	DE	0.990	DE	0.990	DE	0.985	DE	0.9892	6
$X_{2}\cap X_{6}$	0.992	DE	0.989	DE	0.986	DE	0.990	DE	0.989	DE	0.9892	6
$X_{2}\cap X_{8}$	0.994	DE	0.996	DE	0.996	DE	0.995	DE	0.990	DE	0.9942	2
$X_{2}\cap X_{9}$	0.993	DE	0.985	DE	0.989	DE	0.987	DE	0.983	DE	0.9874	9
$X_{6}\cap X_{8}$	0.995	DE	0.996	DE	0.997	DE	0.997	DE	0.984	DE	0.9938	3
$X_{6}\cap X_{9}$	0.991	DE	0.988	DE	0.986	DE	0.993	DE	0.989	DE	0.9894	5
$X_{8}\cap X_{9}$	0.996	DE	0.996	DE	0.996	DE	0.996	DE	0.989	DE	0.9946	1

DE is a two-factor enhancement.

### 5.3. Exploration of influence mechanisms

**Figure 5:**
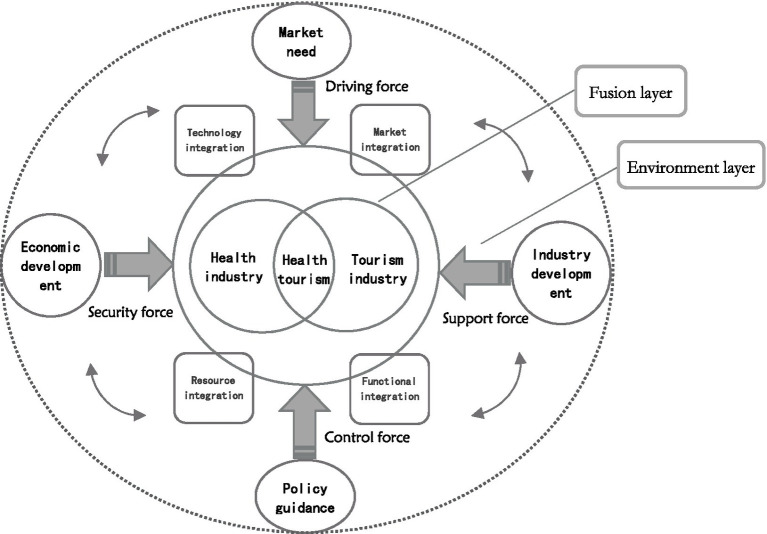
Influence mechanism of the integration of health industry and tourism industry.

The integrated development of industries is a complex systematic project. The health industry and the tourism industry have varied but connected resource conditions, industrial features, and market conditions. Numerous internal and external elements both directly and indirectly influence the integrated development of the two. The influence mechanism framework of the integration of the health industry and tourism industry was built from the perspective of the supply and demand relationship of demand guidance-supply support based on the detection results of the aforementioned influencing factors and combined with pertinent research (see Figure 5). In this framework, stakeholders such as tourists, enterprises, the Chengdu-Chongqing regional government, and the market have different degrees of impact on the integration of the health industry and tourism industry by realizing the coupling of various elements. The impact mechanism consists of four parts. The first is the driving force of market demand. The diversified market demand of tourists for tourism and health has promoted the improvement of the correlation between the tourism industry and the health industry, which is an important driving force for the integrated development of the two industries. The second is the support force of industrial development. The health industry and tourism industry are facing the dilemma of industrial competition and industrial transformation. Only through continuous transformation and upgrading can they better provide products and services that meet consumer needs. The third is policy-oriented regulation. The Chengdu-Chongqing economic circle spans a broad spectrum, and there are variations in economic foundations, resource conditions, policy strength, and administrative systems. Government actions are crucial in removing administrative obstacles, bringing all parties’ interests together, striking a balance between supply and demand, and promoting industrial integration. The fourth is the guarantee of economic development. The interaction between the regional economy and market demand, which is the external driving force for the integration of the two industries, has promoted significant changes in the consumption structure and consumption concept.

In general, the health industry and the tourism industry have a natural coupling, and the two are closely related under the joint drive of internal and external factors, such as policy guidance, economic development, market demand, and industrial development. In the situation of market economy guarantee and government macro-control, the supply and demand coordination role of demand guidance-supply support eventually formed the spatial distribution characteristics of the integration of the health industry and tourism industry in the Chengdu-Chongqing economic circle and further promoted the high-quality integrated development of the two industries through the four integration paths of market integration, resource integration, technology integration, and functional integration.

## 6. Conclusions and recommendations

### 6.1. Conclusions

From a geographic standpoint, the spatiotemporal characteristics of the integration of the health industry and tourism industry in the Chengdu-Chongqing economic circle and its influence mechanism were revealed using the methods of coupling coordination degree, modified gravity model, social network analysis method, and geographic detector. The findings indicated the following:

1. From the standpoint of time series evolution features, the degree of overall coupling coordination of the Chengdu-Chongqing economic circle, the four major regions, and the 36 cities exhibited a fluctuating rising tendency. The Chengdu metropolitan area had a higher level of integration than the other three sectors. Chengdu also had a better coupling effect than Fengdu, which had the worst coupling effect. The integration levels of the other cities in the economic circle were quite low, with the majority of them being less than 0.3 save for the Chengdu and Chongqing main city.
2. From the perspective of spatial differentiation characteristics, the Chengdu-Chongqing economic circle as a whole, the four regional sectors, and the 36 cities were concentrated within the range of (0.1, 0.8), and the regional differences in coupling coordination degree were comparatively large, displaying a three-level ladder. High in the west and low in the east, radiating from the two nuclei to the perimeter were the characteristics of spatial differentiation that were displayed.
3. From the perspective of the characteristics of the spatial connection network, the integration of the two major industries exhibited regional variances in spatial connection strength and established a radial non-equilibrium structure with Chengdu and Chongqing main city serving as connection hubs. This diagram roughly depicted the spatial distribution pattern of two cores high, central weak, and northeast low, which developed into depression in the border areas and traps in the peripheral areas over time. The overall spatial connection network structure was relatively loose, and the point-degree centrality of each city had the spatial distribution characteristics of dominated by two poles, led by multiple cores, and weak at the edge. The cohesive subgroup was greatly affected by geographical factors and administrative divisions.
4. The total income of medical and health institutions was the dominant factor affecting the spatial differentiation of the degree of integration between the two industries. The influence of the two factors under the interaction was greater than that of a single factor. The interaction between the total income of medical and health institutions and the income of health tourism had the strongest explanatory power. The influence mechanism based on the supply–demand relationship of demand guidance-supply support consisted of four parts: market demand driving force, industrial development support force, policy-oriented regulation force, and economic development guarantee force.

### 6.2. Recommendations

The integrated development of the health and tourism industries in the Chengdu-Chongqing economic circle is unbalanced, and regional differences are very apparent as a result of the combined influence of numerous factors, including policy support, market demand, economic development, and industrial development. Attention should be given to the leadership role of the government, erasing administrative divisional barriers, and improving the regional ties between the health industry and the tourism industry in the context of the “Healthy China” strategy, the aging population, and the COVID-19.

#### 6.2.1. Establish a regional coordination mechanism

The government of the Chengdu-Chongqing economic circle should fully understand the temporal and spatial characteristics of the integration of the health industry and tourism industry and speed up the construction of a top-level design that promotes the efficient integration of the health industry and tourism industry. Referring to the legislative achievements of the Chengdu-Chongqing region in the fields of ecological environment, business environment, and transportation construction, focus on the integration status of the two major industries and their spatial relationship is needed to carry out collaborative legislation on healthy tourism and accelerate the establishment of a coupling, coordination, and co-governance mechanism for the health industry and tourism industry in the Chengdu-Chongqing region. Local governments also need to play a cooperative battle, break down the boundaries of administrative divisions, establish a regional coordination mechanism, fill in the depression in the bordering area, and provide an opportunity for cohesive subgroups to generate pairwise spatial connections and form cohesive subgroups that span administrative divisions and geographic locations. All localities must also build a compensation mechanism for synergistic interests; provide policy, financial, and other compensation to the marginal areas of northeastern Chengdu-Chongqing that have fallen into the marginal zone trap in the process of integrated development; and promote the efficient integration and development of the two industries in a mutually beneficial and win-win manner.

#### 6.2.2. Balance the development of the three echelons

Firstly, the first echelon, such as Chengdu and Chongqing main city, has played a radiating role in the health and tourism industry, and strengthened synergy and resource sharing with the second and third echelons by building a cooperation mechanism for health tourism that involves resource sharing, information sharing, mutual promotion of visitor sources and regional linkage. Secondly, the barriers between the four main regions’ administrative divisions in the second echelon should be dissolved. Improving the overall coupling and coordination level of the Chengdu-Chongqing economic circle by optimizing the integration of the four major regional sectors, particularly the southwestern and northeastern parts of Chengdu-Chongqing, must be prioritized. Then transform a single view of health tourism resources and space into a holistic view of health tourism and gradually move closer to the first echelon. The third step is to boost capital investment in the third tier, particularly in the 20 cities around Chongqing, by using financial support and investment promotion strategies. By way of cash transfer payments and other forms of compensation, economic, financial, or policy compensation to third-tier governments whose interests have been harmed throughout the course of regional cooperation should be offered. At the same time, coordinate the layout, branding and marketing of the third echelon of the health tourism industry. Local government departments can jointly carry out health tourism marketing activities, make full use of the “double sun” platform around the region, focus on promoting the region’s health tourism products, enhance their visibility in the tourism market, and thus optimize the coupling coordination level of the third echelon.

#### 6.2.3. Strengthen urban spatial connections

This recommendation comprises the following: Continue to play the role of the two core node cities of Chengdu and Chongqing in the spatial connection network to the surrounding cities. Consider developing node cities like Neijiang, Ziyang, Nanchong, and Rongchang into regional central cities in the southwest Chengdu-Chongqing area, the Chengdu metropolitan area, the northeastern Chengdu-Chongqing area, and the Chongqing metropolitan area. Additionally, improve the pairwise spatial linkages between the cities and the four cohesive subgroups in the spatial connection network while encouraging the in-migration of economic factors, information, and activities into the regional major cities. At the same time, build a transportation network system that integrates land, air and water transportation, establish a land transportation link with high-speed railways as the main link and highways as the supplementary link, build and improve a modern airport network with Chengdu and Chongqing airports as international aviation hubs and airports in medium-sized cities such as Mianyang, Zigong and Luzhou as nodes, combine the special location of the Chengdu-Chongqing region in the middle and upper reaches of the Yangtze River, accelerate the construction of a Yangtze River-based shipping system within the economic circle to minimize the circulation costs of the cities. By expanding the spatial linkage effect of the core cities to the cities in the peripheral areas, it provides an opportunity for the northeastern part of Chengdu and Chongqing to actively seek spatial linkages with the other three geographical segments and to break out of the “peripheral area trap,” thus eliminating the problem of proximity to the group due to the long geographical distance.

#### 6.2.4. Promote synergy of resource elements

The Chengdu-Chongqing economic circle has diverse types of health tourism resources, mainly in the hot spring, forest, sports and ecological categories, and by promoting synergy of resource elements, it will in turn stimulate the recovery of the tourism economy. On the one hand, integrate the health tourism brands. In-depth excavation of the connotation of health culture, around the Ba-Shu cultural tourism corridor, to create cultural IP with Bayu characteristics, highlighting the culture of Chinese medicine and wellness and ethnic minority medicine. The hot spring resources in Chongqing should promote the deep integration of the tourism industry and the hot spring health and wellness industry, with the hot spring health and wellness tourism as the focus, highlighting the title of “World Hot Spring Capital” and forming an international brand of hot spring health and wellness tourism. The Sichuan area is rich in forest resources, and the integration of the tourism industry with the forest health and wellness industry should be promoted in-depth, and well-known brands of forest health and wellness tourism should be cultivated. On the other hand, integrate and plan the health tourism spaces. The Chengdu-Chongqing regional government should conduct field research with enterprises and experts, handle the relationship between points, lines and surfaces, and plan a circle-level development model by combining the characteristics of the spatial linkage network, with the main cities of Chengdu and Chongqing main city as large circles and the sub-node cities in the same cohesive sub-group, such as “Chengdu, Deyang, Meishan, Ziyang” and “Suining, Luzhou, Hechuan, Qijiang” as small circles, with the large circles driving the small circles and the small circles driving the common development of the surrounding areas.

### 6.3. Research contribution

Firstly, the spatial and temporal characteristics of the integration of the health and tourism industries were analyzed in two dimensions: chronological and spatial evolution, In particular, it portrays the characteristics of the network of spatial links between the integration of the health industry and the tourism industry based on the perspective of industrial integration. The use of spatial linkage networks to portray the spatial characteristics of the integration of the health and tourism industries is complementary to existing research. Based on the characteristics of industrial integration, this study introduces the coupling and coordination degree of the two industries to modify the traditional Newtonian gravitational model. The modified gravitational model can construct the spatial linkage network of the integration of the health industry and tourism industry relatively accurately, and better reflects the spatial characteristics of the integration of the health industry and tourism industry.

Secondly, the framework of the influence mechanism of the integration of the health and tourism industries is constructed from the perspective of supply and demand. Previous studies have mostly analyzed qualitatively the factors affecting the integration of the health and tourism industries, without further exploring the degree of influence of each factor, and in the previous literature, economic factors are usually more influential than policy factors and are the dominant factors affecting the integration of the two industries. This study adds two innovative indicators to the index system of influencing factors, namely the number of health tourists and health tourism revenue, to quantitatively measure the influence of each factor on the integration of the two industries, and concludes that the influence of policy factors is greater than that of economic factors, which is different from previous studies. It also constructs a framework for the influence mechanism of the integration of the health industry and the tourism industry from the perspective of supply and demand, which is a useful supplement to the existing research results.

In short, the high-quality integrated development of the health industry and the tourism industry is a complex systematic project, which requires the assistance of government departments at all levels and health tourism enterprises in the Chengdu-Chongqing economic circle to strengthen coordination and cooperation. However, due to the wide coverage of the Chengdu-Chongqing economic circle and difficulty in obtaining data, there were certain limitations in the selection of indicators, which could not fully reflect the temporal and spatial characteristics of the two major industries and their influencing factors. The limitations of the data make this study’s understanding of the integration of health and tourism industries in the Chengdu-Chongqing economic circle only in the last 5 years, and there is a lack of horizontal comparison of the effects of the integration and development of the two industries at the regional level, such as the Yangtze River Delta and the Pearl River Delta. The research method used is also more traditional and does not directly reflect the spatial differentiation of the integration of the two industries in the Chengdu-Chongqing economic circle. Therefore, we can rely on the cultural and tourism committees in Chengdu and Chongqing to obtain relevant data and conduct in-depth field research to measure the coupling relationship between the two and the influencing factors based on the characteristics of the health and tourism industries with newer research methods and more comprehensive indicators. A side-by-side comparison of the effects of the integration of the health and tourism industries in the Chengdu-Chongqing economic circle of the Yangtze River Delta and the Pearl River Delta with the Chengdu-Chongqing region can also be developed at the regional level, analyze the advantages and disadvantages of the integrated development of the two industries in the Chengdu-Chongqing region through horizontal comparison, and draw on the useful experience of other regions in the integration of the health and tourism industries. In addition, this study found that policy support is the dominant factor influencing the integration of the health and tourism industries, but the extent to which policies can promote the integration of the two, how effective their implementation is, and what impact a combination of policies at national and local levels will have on the two industries are all areas that future research can continue to focus on.

## Data availability statement

The datasets presented in this study can be found in online repositories. The names of the repository/repositories and accession number(s) can be found in the article/supplementary material.

## Author contributions

XC is responsible for formulating research ideas and research design, and revising and improving the thesis. YD is responsible for literature analysis, thesis writing, and graph drawing. All authors contributed to the article and approved the submitted version.

## Funding

This work was funded by Chongqing Social Science Planning Excellence Program Project “Research on the Innovative Development of Chongqing Health Tourism Industry in the New Era” (2021YC046); Sichuan Tourism Development Research Center, a key research base of philosophy and social sciences in Sichuan Province, “Research on Collaborative Innovation Development of Health Tourism in Chengdu-Chongqing Economic Circle” (LY22-05).

## Conflict of interest

The authors declare that the research was conducted in the absence of any commercial or financial relationships that could be construed as a potential conflict of interest.

## Publisher’s note

All claims expressed in this article are solely those of the authors and do not necessarily represent those of their affiliated organizations, or those of the publisher, the editors and the reviewers. Any product that may be evaluated in this article, or claim that may be made by its manufacturer, is not guaranteed or endorsed by the publisher.
